# Development and Characterization of Biodegradable, Binderless Fiberboards from Eggplant Straw Fibers

**DOI:** 10.3390/ma18010037

**Published:** 2024-12-25

**Authors:** Hailun Fan, Xiulun Wang, Tingting Wu, Jianzhong Sun, Jun Liu

**Affiliations:** 1 Graduate School of Bioresources, Mie University, Tsu 514-8507, Japan; 523d2s2@m.mie-u.ac.jp; 2 International Joint Research Center on High-Value Utilization of Agricultural Waste Biomass Between Jiangsu University, China, and Mie University, Japan, Zhenjiang 212013, China; jzsun1002@ujs.edu.cn (J.S.); junliu115142@hotmail.com (J.L.)

**Keywords:** fiberboard, binder-free, bio-board, biodegradable eggplant straw, rupture stress

## Abstract

Currently, wood-based panels are mainly made from wood and adhesives containing formaldehyde. With the growing demand for raw materials and increasing concern for human health, the use of residues from annual crops to manufacture binder-free biodegradable biomass boards has attracted increasing interest. The aim of this study was to develop a biodegradable bio-board without any adhesives using eggplant straw fibers. The bio-boards were produced via simple mechanical refinement of eggplant straw fibers and were formed under pressures of 2.0 MPa, 3.5 MPa, 5.0 MPa, 6.5 MPa, and 8.0 MPa. The mechanical properties and dimensional stability of the manufactured bio-boards were evaluated. With increasing applied pressure, the bending rupture stress of the bio-boards increased from 27.69 MPa to 45.29 MPa, the tensile rupture stress varied from 12.45 MPa to 24.62 MPa, the water absorption decreased from 91.45% to 88.29%, and the contact angle increased from 89.67° to 90.45°. The bio-boards were subjected to morphological analysis (SEM) and porosity and crystallinity measurements (XRD), and the results indicated that the water absorption of the bio-boards was due to a combination of porosity and crystallinity. The results showed that eggplant straw is suitable for manufacturing bio-boards.

## 1. Introduction

According to statistics, more than 11.9 billion tons of various grains and vegetables are produced globally every year [1]. Straw as a by-product of grains and an inedible part of vegetables, is produced in large quantities at the same time during food production. He et al. [2] pointed out that the straws of grains and inedible parts of vegetables from the food production process are often landfilled or incinerated, which negatively impacts the environment. In recent years, achieving carbon neutrality, with a focus on energy conservation, emission reductions, and sustainable development, has become important [3]. Therefore, how to effectively utilize these agricultural wastes and realize the sustainable development goals (SDGs) has become one of the most pressing issues. Although progress has been made in the utilization of agricultural waste, large quantities of grain straw and inedible parts of vegetables have not been effectively utilized. Grain straw and inedible parts of vegetables, which are plant biomass, are renewable biomass resources. The main components of plant biomass are cellulose, hemicellulose, and lignin [4]. Cellulose is one of the most abundant organic compounds on earth, and the annual production of cellulose is estimated to be 1.5 × 1012 tons [5]. Owing to their biodegradability and renewability, cellulosic materials have attracted much attention and are the most promising candidates for cellulose-based polymer materials [6]. Cellulosic materials play an indispensable role in daily life because of their excellent properties, such as low carbon emission, low cost, and good mechanical properties. They are widely used in various fields such as paper making, textiles, building materials, packaging, and biofuels [7,8]. The use of cellulosic materials to produce binderless fiberboard was first advocated in the 1980s. Initial attempts to utilize industrial bagasse waste to produce composite materials via self-bonding technology were made [9]. Currently, approximately 95% of the lignocellulosic material used for particleboard production is wood [10]. With the rapid development of the global economy, the demand for wood-based products such as particleboard continues to rise; therefore, concerns about the depletion of wood resources are increasing [11]. In the current particleboard manufacturing process, formaldehyde-based chemical adhesives need to be added, and the formaldehyde released from particleboard is harmful to human health [12]. To address the above problems, a fiberboard manufacturing process that uses annual plants as raw materials must be developed, and fiberboard manufacturing methods that do not use chemical adhesives must be explored [13].

In recent years, the production of fiberboards using non-woody raw materials has attracted much attention. In this context, various non-woody materials such as perennial plants (sisal, hemp, flax, kenaf, jute, and ramie) have been used since ancient times [14], and agricultural residues (e.g., corn straw, rice straw, wheat straw, sorghum straw, soybean straw, tomato straw, and eggplant straw) [15,16], and grasses (e.g., African couch grass, Napier grass, graviola grass, and energy grass) [17,18,19] have also been proposed as sources of fiber material in recent years. Fiber materials are also available in marine biomass such as seaweed and seagrass [20,21].

Eggplant (*Solanum melongena*) originated in India, then gradually spread to China and Japan before spreading to the Mediterranean region, Africa, Europe, and America, where it is now cultivated worldwide. As a vegetable, eggplant is widespread, with an annual production of more than 50 million tons [22], and is one of the seven most consumed vegetables in the world. The stalks of eggplants are agricultural residues and are an underutilized biomass material. These stalks are rich in cellulose fibers and can be considered good raw materials for the manufacture of bio-boards and other [23,24].

Formaldehyde-based resins are used as binders in conventional wood-based panels to form and improve the structural strength and stability of the material. Formaldehyde is a component of some adhesives (e.g., phenolic resins and urea–formaldehyde resins). It is often used in the formation of wood-based materials because of its low cost and desirable properties. However, owing to its volatility, exposure to high levels of formaldehyde for long periods can cause health problems in humans [25]. As the cost of adhesives continues to rise in the international market, the commercial price of wood-based panels is also increasing, which gradually reduces the attractiveness of wood-based panels [26]. In addition, most adhesives are generally non-renewable and non-biodegradable, which causes great difficulties in recycling wood-based panels. The mechanical properties and dimensional stability of bio-boards can be improved by using natural binders (soybean-based binders, lignin, tannin adhesive, etc.) instead of formaldehyde-based resins or by physically, chemically, or biologically treating the material; however, these processes usually require more complex processing steps and entail higher economic costs [27,28]. In summary, to reduce manufacturing costs and save energy while enhancing the applicability of bio-boards, it is crucial not only to identify manufacturing processes that meet both standards and economic efficiency within reasonable conditions but also to eliminate the need for any chemical adhesives. By relying solely on the hydrogen bonding between cellulose fibers, a more efficient, economical, and environmentally friendly production method can be achieved.

The purpose of this study is to develop a biodegradable biomass board (hereafter, biodegradable biomass board will be referred to as bio-board) using non-edible parts of eggplant without any adhesives. The bio-board can be applied in many fields instead of fiberboard, which is produced with chemical adhesives. Bio-boards manufactured under various applied pressures were investigated from the micro and macro perspectives. Regarding the microscopic aspect, the morphological properties and crystallinity of the bio-boards produced under different applied pressures were examined via scanning electron microscopy (SEM) and X-ray diffraction (XRD). Regarding the macroscopic aspect, the bio-board samples were tested for their mechanical properties and water resistance. The effects of the applied pressure on the mechanical properties of the bio-boards and the causes of variations in the water absorption of bio-boards were investigated. In addition, the research results were compared with those from studies on adhesive boards produced by other researchers. The possibility of using bio-boards as the replacements for existing wood-based panels with adhesives is verified, and this research also provides a new way of using non-edible parts of vegetables as biomass materials.

## 2. Materials and Methods

### 2.1. Material

Eggplant straws (excluding eggplant leaves), as the raw material for this study, were collected from Mie Prefecture, Japan, and the variety is PC Chikuyo, which is the most common variety of eggplant in Japan. The dried straws were shredded into pieces less than 10 mm in size using an electric grinder (SU16; Cowa Cutter Co., Shizuoka, Japan). The eggplant straw pieces were stored at a temperature of 20 ± 1 °C and a relative humidity of 65 ± 5% for further use.

### 2.2. Chemical Characterization of the Raw Material

The chemical composition is important for producing binderless fiberboard. Owing to the lack of a binder, each component of eggplant straw has a greater effect on the bio-board properties. The chemical composition of raw materials varies depending on the type of material and different parts of the plant [29]. Since eggplant straw is a heterogeneous material, to accurately analyze the chemical composition, various parts of eggplant straw were ground into 40 mesh fine particles, then evenly mixed. To assess cellulose, hemicellulose, and lignin contents, Van Soest and Wine’s ADF/NDF method was adopted [30,31]. All analyses were carried out in triplicate.

### 2.3. Manufacturing of Bio-Boards

**Figure 1:**
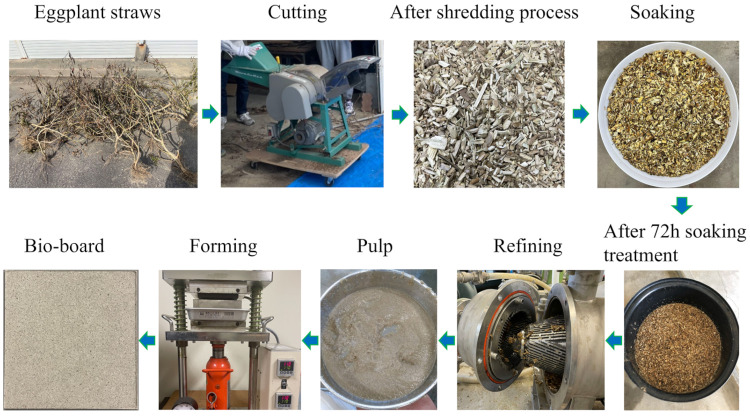
Making process of bio-board.

In this work, a wet process method was employed to produce bio-boards without the use of any chemical additives. Figure 1 outlines the experimental sequence for the fabrication of the self-bonded cellulose material. Prior to manufacturing the bio-board, fine pieces of eggplant straw were soaked in water at a temperature of 20 ± 1 °C for 72 h to soften them. The raw material was then mechanically refined using a pulper (Model A Beat finer; Satomi Corp., Shizuoka, Japan), with fibers defibrating in the presence of a large amount of water (94–96%). For this purpose, 1 kg of eggplant straw was mixed with 20 L of water, and the defibrated fibers were passed through 0.25–4.00 mm sieves. The defibrated pulp was then filled into a mold and pressed. Hydrogen bonds form between the pulp fibers and water, and as the water is removed, the distance between the fibers decreases. The fibers then bond to each other through hydrogen bonds, resulting in the formation of a bio-board. To remove the water from the pulp, pressure and temperature were applied to the mold via a hot presser (IMC-180C; Imoto Machinery Co., Kyoto, Japan). A bio-board with a square size of 100 mm × 100 mm was manufactured. The conditions for manufacturing the bio-boards are shown in Table 1. To control the mass of the bio-boards at approximately 20 g, the amount of pulp used was adjusted.

### 2.4. Pre-Conditioning of Samples Before Testing

In this study, all bio-board samples were pretreated in accordance with the JIS A5905:2014 standard (Japan Standards Association 2014) [32]. Prior to the experiment, the samples were subjected to pre-treatment at a temperature of 20 ± 2 °C and a relative humidity of 65 ± 5% until they reached a constant weight. A constant weight is defined as the weight measured every 24 h, with a rate of change of 0.1% or less. The samples were treated for three days, after which a constant weight was reached.

### 2.5. Scanning Electron Microscopy Micro Analysis

The surface morphology and internal structure of the bio-boards were measured via SEM. The bio-boards were cut into 5 mm × 5 mm samples using an ultrasonic cutter (ZO-40B; Honda Plus, Shinshiro, Japan) and these samples were used to observe the surface morphology of the bio-boards. Directly broken bio-board samples were obtained to investigate the internal structure. A high-resolution ion sputterer (208HR, Cressington, UK) was employed to deposit a thin layer of gold onto the surface and cross-section of the test piece, followed by measurement of the bio-board microstructure and morphology using a scanning electron microscope (Apreo 2C, Thermo Fisher Scientific, Waltham, MA, USA). The scanning electron microscope was operated at an acceleration voltage of 5.00 kV with a magnification of 500×.

### 2.6. X-Ray Diffraction Analysis

A 25 mm × 25 mm sample was initially cut from the bio-boards using an ultrasonic cutter and analyzed with an X-ray diffractometer (Uitima IV, Rigaku, Japan) to assess the crystallinity of the bio-boards manufactured under different pressures. X-ray diffractograms were obtained using Cu-Kα radiation (λ = 1.5406 Å) by placing the sample on a block sample holder. The X-ray tube was a copper tube with a tube potential of 40 kV and a tube current of 40 mA, and a continuous 2θ/θ scan was performed. The slit device was set to DS = 0.5°, SS = open, and RS = 8 mm. The scanning speed was set to 1°/min, and the scanning step angle was 0.02 degrees. The crystallinity curves for each condition (2.0 MPa to 8.0 MPa) were fitted using open-source software (Jade 6), and the iterations were repeated to achieve an R2 value of 1.37% for each condition.

### 2.7. Measurement of Physical Characteristics

To investigate the fundamental physical properties of the bio-boards, the porosity, density, and moisture content of the bio-boards needed to be measured. In this study, we measured the porosity of the bio-boards manufactured under different fabrication conditions via the gas displacement method using an SC-4000T (Xiamen San Chuang Science Instrument Testing Equipment Co., Ltd., Xiamen, China) equipped with a 20 cm^3^ chamber. The instrument was pre-treated three times before each test to ensure the accuracy and repeatability of the measurement results. Moreover, to prevent measurement errors, sixteen 25 × 25 mm bio-board samples were used to measure the porosity. The measurement of each bio-board sample was repeated eight times. Additionally, the apparent density of the bio-boards was determined by measuring their mass and volume. The moisture content of the bio-boards was calculated by measuring the sample weight before and after drying. The bio-board samples were dried at 110 °C for 24 h in a dryer.

### 2.8. Analysis of the Mechanical Properties

To evaluate the strength properties of the bio-boards, the bending, tensile, and screw holding force tests were carried out using a universal testing machine (SVZ-200NB-200R2, IMADA Corp., Toyohashi, Aichi, Japan) according to the JIS A5905:2014 standard [32]. Six test pieces from bio-boards manufactured under the same conditions were tested, and bending stress, tensile stress, and screw holding force were measured. For the bending test, rectangular specimens with dimensions 20 × 50 mm were used, whereas dumbbell-shaped specimens were used for the tensile test, and square specimens with dimensions 25 × 25 mm were used for the screw holding force test. The three-point bending test was performed using a span of 40 mm between the two supports. The loading speed was set at 15 mm/min for the bending test and 2.4 mm/min for the tensile and screw holding force tests.

### 2.9. Analysis of the Dimensional Stability

The dimensional stability of the bio-boards was assessed by conducting water absorption and surface wettability tests. Four 50 × 50 mm samples were used to evaluate the length change (LE), thickness swelling (TS), and water absorption (WA) characteristics of the bio-boards. The samples were immersed in water and placed in a controlled environment incubator (PR-1KPH, ESPEC, Osaka, Japan) maintained at a temperature of 20 ± 0.3 °C and a relative humidity of 65 ± 2.5% RH for 24 h. Subsequently, precise measurements of the length and thickness were taken at predetermined locations to calculate the extent of swelling. To determine the water absorption, each sample was carefully weighed before and after immersion, with an electric scale accuracy of 0.001 g.

To assess the surface wettability, a contact angle test was conducted. A small droplet of distilled water (5 μL) was deposited onto the surface of the bio-board. High-resolution digital microscopy (NDL-40Z; Hirox Corp., Tokyo, Japan) was employed to capture an image of the droplet, and the contact angle between the droplet and the bio-board surface was determined using the open-source software ImageJ 1.54d. This analysis provided a valuable index for the hydrophobicity or hydrophilicity properties of the solid surface.

### 2.10. Statistical Analysis

All of the bio-board characterization data were analyzed five times, and the obtained data were subjected to statistical analysis via one-way analysis of variance (ANOVA). Statistical significance was determined at a threshold of *p* < 0.05.

## 3. Results and Discussion

### 3.1. Characterization of Eggplant Straw

The chemical composition of eggplant straw was determined via the method described above. The results in Table 2 are compared with those of non-woody and woody lignocellulosic materials used in other studies for the production of fiberboards [33,34]. The chemical composition results are consistent with those reported in previous studies on eggplant straw. The cellulose content of eggplant straw is almost identical to that of rice straw and differs by only 6% from those of tomato straw and camphor. The lignin content is twice as high as that of tomato and slightly lower than those of rice straw and camphor. The hemicellulose content is greater than those of rice straw and tomato straw, but lower than that of camphor. This comparison reveals that eggplant straw has moderate lignin and hemicellulose contents and similar cellulose contents, making it a suitable alternative source to woody and non-woody materials for use in bio-board manufacturing.

### 3.2. Analysis of the Morphology of the Bio-Boards (SEM Analysis)

**Figure 2:**
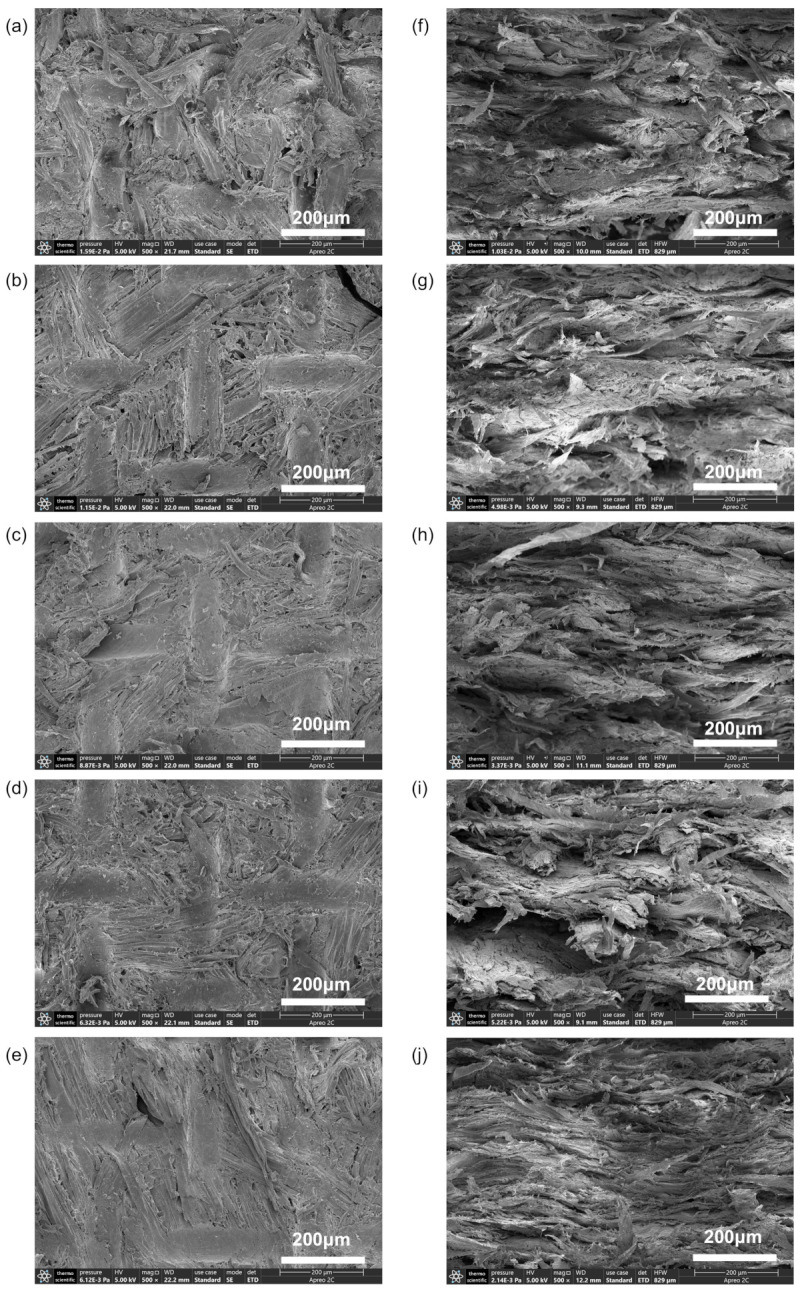
(**a**–**e**) Surface images of bio-board samples produced at 2.0–8.0 MPa, (**f**–**j**) Cross-sectional images of bio-board samples produced at 2.0–8.0 MPa.

From a macroscopic point of view, considering that no adhesive was added to the bio-boards, the surface of the bio-boards did not show any signs of debris shedding, which indicates a relatively strong bond between the fibers. The surface morphology of bio-boards manufactured under applied pressures ranging from 2.0 to 8.0 MPa was examined via SEM at a viewing scale of 200 μm. The measurement results are shown in Figure 2a–e, many tubular pores exist on the fibers. In addition, because the arrangement of the natural fibers is disordered, many gaps exist between the fibers. These tubular pores and gaps are expected to accommodate a large amount of air and water molecules. However, as the applied pressure increases, the fiber distribution becomes more uniform and the gaps on the surface of the bio-board gradually decrease, which can reduce the entry of external water and improve the water resistance of the bio-board. As the pressure applied during the manufacturing process of the bio-boards increases from 2.0 MPa to 3.5 MPa, the gaps between the fibers significantly decrease. When the pressure exceeds 3.5 MPa, the magnitude of the change becomes negligible.

The internal bonding characteristics of the bio-boards can be clearly observed via SEM at an observation scale of 200 μm (Figure 2f–j). The thermoforming and pressure promote defibration of the eggplant straw fibers and create a bonding effect. Fiber bundles are cross-linked and entangled with each other, forming multiple laminar structures. These multiple laminar structures lead to a tight connection between the fibers that gradually strengthens the interwoven fibers and makes them denser [35]. This compact structure increases the density and mechanical strength of the bio-board. The dense cross-sectional layered structure also inhibits water infiltration into the bio-board, thereby increasing the water resistance of the bio-board [36].

### 3.3. X-Ray Diffraction

**Figure 3:**
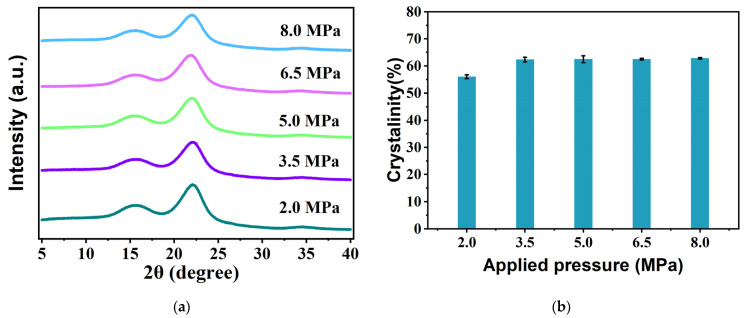
XRD results and crystallinity of bio-board samples produced at different pressures (*p* < 0.05). (**a**) XRD results of bio-board samples; (**b**) crystallinity of bio-boards.

The mass fraction of crystalline regions in cellulosic materials plays a pivotal role in their chemical, mechanical, and physical properties. Figure 3a,b show XRD patterns of the bio-boards manufactured under various pressures, accompanied by a comparison of their crystallinity. The major diffraction peaks at 2θ = 14.8° (−110), 16.3° (110), 22.6° (200), and 34.6° (004) correspond to cellulose type I [37]. The peak regions and other areas are categorized as crystalline and non-crystalline regions, respectively. Cellulose type I encompasses a combination of two distinct crystalline forms: cellulose Iα (trigonal crystalline system) and Iβ (monoclinic crystalline system). Cellulose type I crystals form through molecular hydrogen bonding within the cellulose matrix, thereby contributing to their stability. Prior studies have demonstrated that a high crystallinity signifies a tightly packed arrangement of fiber chains, which is crucial for achieving enhanced mechanical strength, density, and dimensional stability [38,39]. Notably, our observations reveal an increase in the crystallinity with increasing pressure. However, remarkably, the change in the crystallinity becomes negligible once the pressure reaches 3.5 MPa. Within the pressure range of 3.5–8.0 MPa, the increase in the crystallinity is only 0.44%. Furthermore, the crystallinity of the bio-boards produced at 2.0 MPa differs by 6% from that of the bio-boards produced under the other pressure conditions. These results indicate a gradual stabilization of the bio-board structure beginning at 3.5 MPa. Additionally, these findings align with the SEM images depicted in Figure 2, which illustrate minimal surface alteration of the bio-boards between 3.5 MPa and 8.0 MPa. These discoveries provide valuable insights into the physical and mechanical properties of the bio-boards.

### 3.4. Physical Properties

**Figure 4:**
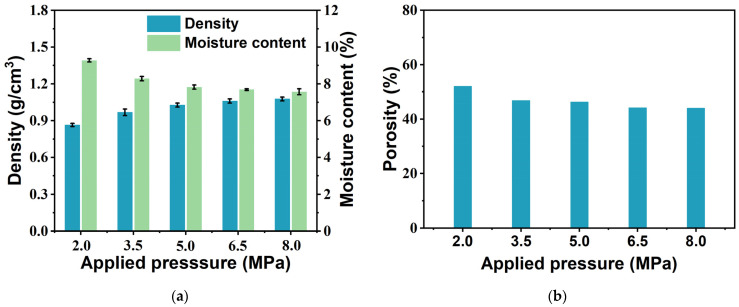
Density, moisture content, and porosity of bio-board samples produced at different pressures. (*p* < 0.05) (**a**) Density and moisture content; (**b**) porosity.

To evaluate the physical properties of the bio-boards, the density, moisture content and porosity of the bio-boards manufactured under different pressures were measured and are shown in Figure 4a. The density of the bio-boards varies between 0.87 g/cm^3^ and 1.08 g/cm^3^ with increasing pressure, and the moisture content ranges from 9.27% to 7.57%. These results meet the requirements of JIS A5095 for hardboard, i.e., a density of more than 0.8 g/cm^3^ and a moisture content between 5% and 13% [32]. When the applied pressure increased from 3.5 MPa to 8.0 MPa, the density of the bio-board produced at 8.0 MPa increased by 11% relative to that at 3.5 MPa, while the moisture content decreased by 8.6%. In addition, as shown in Figure 4b, the porosity of the bio-boards decreases from 52.09% to 44.03% with the increasing applied pressure. The porosity of the bio-board produced at 8.0 MPa was reduced by 6% relative to that at 3.5 MPa. The trend of the density is inversely proportional to that of the porosity increasing applied pressure. SEM images confirm this phenomenon. As the applied pressure increases, the fibers become more tightly bound to each other, thus reducing the number of tiny voids and expelling air and water molecules from the voids [40].

### 3.5. Mechanical Properties of the Bio-Boards

**Figure 5:**
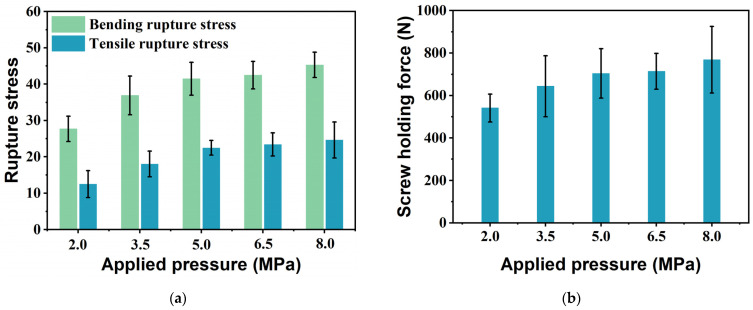
Rupture stress and screw holding force of bio-board samples produced at different pressures. (**a**) Bending rupture stress and tensile rupture stress; (**b**) screw holding force. (*p* < 0.05).

To investigate the mechanical properties of the bio-boards, bending, tensile, and screw holding force tests were carried out. The bending rupture stress and tensile rupture stress of the bio-boards were calculated and are shown in Figure 5a. The bending rupture stress and tensile rupture stress increase with increasing applied pressure. The bending rupture stress changed from 27.69 to 45.29 MPa. The maximum bending rupture stress was 45.29 MPa at 8.0 MPa of applied pressure. The tensile rupture stress of the bio-boards accordingly increases, increasing applied pressure. The tensile rupture stress changed from 12.45 to 24.62 MPa. The maximum tensile rupture stress was 24.62 MPa at an applied pressure of 8.0 MPa. When the applied pressure was increased from 3.5 MPa to 8.0 MPa, the bending fracture stress of the bio-boards produced at 8.0 MPa increased by 22.8% relative to the bending rupture stress at 3.5 MPa, while the tensile rupture stress increased by 36.7%. The bio-boards produced under applied pressures ranging from 3.5 MPa to 8.0 MPa satisfy the requirements for S35-type hard fiberboard, as specified by the JIS A5905 2014 standard [32]. The distance between the fibers decreases with increasing applied pressure, which promotes the formation of hydrogen bonds and enhances the mechanical properties of the bio-board. In general, the strength is consistent with the crystallinity, and the rupture stress is consistent with XRD analysis results in this study but is not a perfect match. From the applied pressure of 3.5 MPa onward, the increasing rate of the crystallinity of the bio-boards slows, whereas the change in the strength is relatively obvious. For non-homogeneous materials, many factors affect the mechanical properties of bio-boards; for example, the size and orientation of fibers can influence their mechanical properties [41,42].

In practical applications, furniture and building materials are usually installed using screws for fastening, so evaluating the screw holding force of the bio-boards is crucial. Figure 5b shows the screw holding force of bio-boards. The screw holding force increased with increasing applied pressure, and varied from 541.38 N to 768.59 N. The screw holding force of the bio-board produced under an applied pressure of 8.0 MPa increased by 17.1% relative to that of the bio-board produced under 3.5 MPa. In general, the higher the density is, the stronger the internal bonding force, and therefore the higher the resistance when the screw is pulled out from the bio-board. The maximum density of the bio-boards is obtained at the maximum applied pressure, and the maximum screw holding force of 768.59 N occurs at the maximum density of the bio-boards. The trend of the screw holding force of the bio-boards is similar to that of the bending rupture stress.

The mechanical properties (bending rupture stress, tensile rupture stress, and screw holding force) of the bio-boards manufactured in this study are superior to those of commercially available fiberboards [43,44]. This demonstrates the possibility of utilizing bio-boards instead of commercially available fiberboards.

### 3.6. Hydrophobicity and Water Resistance

**Figure 6:**
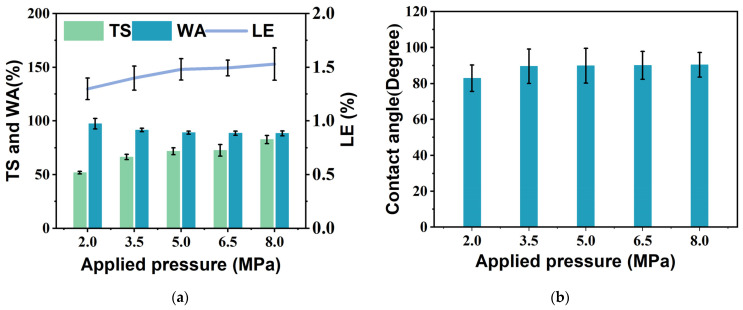
WA, TS, LE, and contact angle of bio-board samples produced at different pressures. (**a**) Water absorption (WA), thickness swelling (TS), and linear expansion (LE); (**b**) contact angle. (*p* < 0.05).

Cellulose-based materials have a high sensitivity to moisture, which may reduce their service life in durable projects [45]. After the bio-boards were immersed for 24 h according to JIS A5905 [32], none of the surfaces of the bio-boards were cracked, and no fiber residue appeared in the water. The water absorption and change in the length and thickness expansion are shown in Figure 6. The rate of change in the length and thickness expansion of the bio-board increased with increasing applied pressure in contrast to the water absorption. The maximum thickness expansion of the bio-board was 82.67% at 8.0 MPa, which is 59.5% greater than the minimum thickness expansion of 51.84% at 2.0 MPa. The length variation of the bio-boards ranged from 1.30% to 1.51%. The water absorption of the bio-boards ranged from 88.29% to 97.32%. The thickness expansion rate of the bio-boards remarkably increased, whereas the length change rate and the water absorption rate changed more slowly. The water absorption rate of the bio-board produced under an applied pressure of 8.0Pa decreased by 3.5% relative to that produced under 3.5 MPa. In contrast, the length change rate increased by 7.7% and the thickness expansion rate increased by 24.5%. The water absorption rate did not change significantly.

**Figure 7:**
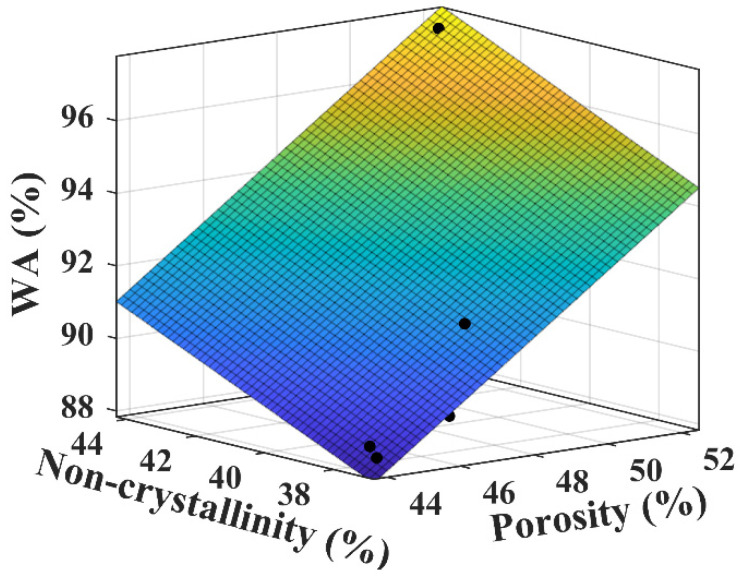
Correlation analysis between water absorption, porosity, and non-crystallinity.

Plywood or wood building materials contain many lignocellulosic components (fibers) that are usually hydrophilic when in contact with water. The macromolecular single bonds of the fiber cell wall contain many OH bonds, and upon contact with water, new hydrogen bonds are formed between the H groups of the water molecules and the OH groups of the single bonded fiber matrix. These interactions lead to swelling of the fiber matrix and weakening of the interfacial bond strength, which results in matrix cracking and dimensional instability [46]. As the applied pressure increases, the distance between the fiber layers decreases, the number of hydrogen bonds increases, and the fiber layers become more tightly bound to each other, a situation that limits the entry of water, which leads to a decrease in water absorption. The moisture content of the fibers depends on the non-crystalline part content and void content of the fibers. In this study, the trends of the changes in the cellulose crystallinity and porosity stabilize at an applied pressure of 3.5 MPa. This phenomenon indicates that under this pressure, the molecular arrangement in the cellulose structure has reached a stable state, and a further increase in the pressure will not significantly change its crystallinity or porosity. This stable change trend is also reflected in the overall crystallinity of the bio-boards, which means that the structure of the material has become stable, and the arrangement of the internal fibers no longer changes significantly changes, resulting in the water absorption rate gradually becoming stable. To this end, a multiple linear regression analysis was conducted to examine the correlation between the water absorption of the bio-board and its porosity and non-crystallinity, resulting in the regression equation: f (x, y) = 0.76x + 0.43y + 38.2. In the multiple regression analysis, the adjusted R^2^ was used to evaluate the goodness of fit, yielding an R^2^ value of 0.92. The statistical indicators indicate a high level of regression correlation. Water absorption is a linear function related to porosity and non-crystallinity, where the coefficient for non-crystallinity (0.43) is significantly smaller than that for porosity (0.76), suggesting that changes in porosity have a greater impact on water absorption. Figure 7 shows the comparison between the regression model and the experimental data, and the residual analysis indicates that the model fits the observed data well, further validating the correlation between water absorption, porosity, and non-crystallinity. These results demonstrate that, within the pressure range of 2.0 MPa to 8.0 MPa, both porosity and non-crystallinity have a significant effect on the water absorption of the bio-board.

A contact angle analysis enhances our understanding of the hydrophilicity of the bio-boards based on the measured angle between water droplets and the bio-boards. A contact angle of less than 90 degrees (<90°) indicates hydrophilicity, or poor water resistance, whereas a contact angle of more than 90 degrees (>90°) indicates hydrophobicity, or high water resistance [47]. Figure 6b shows the contact angle of the bio-boards. The contact angle increased with increasing applied pressure. The contact angle ranged from 82.93° to 90.45°. The maximum contact angle was 90.45° at the maximum applied pressure of 8.0 MPa, and the bio-board reached a minimum contact angle of 82.93° at 2.0 MPa. Notably, the contact angle of the bio-board produced under an applied pressure of 8.0 MPa increased by 0.87% compared to that of the bio-board produced under 3.5 MPa; this change in contact angle is extremely small. SEM analysis revealed that the surface morphologies of the bio-boards are almost identical starting from an applied pressure of 3.5 MPa, providing further evidence for the change in the contact angle.

### 3.7. Energy Savings and Economy

Regarding bio-board, the standards mentioned vary depending on the application. For example, in the case of JIS A5905 2014 for hardboard, the density, water content, and mechanical properties of bio-boards already satisfy the standards for S35-type hardboard at a manufacturing pressure of 3.5 MPa. At the same time, the change in water absorption and contact angle from 3.5 MPa to 8.0 MPa is minimal. When the manufacturing pressure of the bio-board was increased from 3.5 MPa to 8.0 MPa, the changes in the physical properties were minimal and the improvement in the mechanical properties was relatively limited despite the 128.6% increase in pressure. Since the manufacturing process of the bio-boards was manually operated, it was not possible to accurately assess the energy savings. However, from an energy saving and economic point of view, this means that no additional energy is required to enhance the performance of the bio-board. Therefore, the 3.5 MPa manufacturing condition realizes the economy of the bio-board.

## 4. Conclusions

In this study, a resource-efficient utilization approach to manufacture binder-free and biodegradable biomass boards from eggplant straw is proposed, which is in line with the concept of sustainable development. The bio-boards manufactured in this study have good performance; in particular, bio-boards manufactured at an applied pressure of 8.0 MPa have excellent mechanical properties. However, from the perspective of energy savings and cost efficiency, an applied pressure of 3.5 MPa is more appropriate, as the biomass board not only satisfies the requirements for S35-type hardboard but also provides significant energy savings. Additionally, the analysis of the bio-boards in terms of XRD and porosity results provides an in-depth explanation of the variation in the water absorption of the bio-boards. However, even after achieving the best performance at 8.0 MPa, the bio-board still suffers from excessive water absorption. Furthermore, the highest applied pressure does not reach the limit of material performance. Therefore, reducing the water absorption of bio-boards and applying higher pressures to produce great high-performance bio-boards should be considered important goals for future research.

## Figures and Tables

**Table 1:** Experimental conditions for manufacturing bio-boards.

Applied Pressure (MPa)	Heating Temperature (°C)	Heating and Pressing Time (min)
2.0, 3.5, 5.0, 6.5, 8.0	110	120

**Table 2:** Comparison with other materials.

Raw Materials	Cellulose (%)	Hemicellulos (%)	Lignin (%)
Eggplant straw	37.17	16.45	18.08
Rice straw	37.70	7.20	27.90
Tomato stalk	43.11	7.91	12.29
Camphor tree	43.00	22.70	25.10

## Data Availability

The raw data supporting the conclusions of this article will be made available by the authors on request due to this study is still in progress.
